# Bicuspid aortic valve disease: systematic review and meta-analysis of surgical aortic valve repair

**DOI:** 10.1136/openhrt-2016-000502

**Published:** 2016-12-16

**Authors:** Maximilian Salcher, Huseyin Naci, Sarah Pender, Titus Kuehne, CARDIOPROOF Consortium, Marcus Kelm

**Affiliations:** 1 Department of Social Policy, LSE Health, London School of Economics and Political Science, London, UK; 2 London School of Hygiene and Tropical Medicine, London, UK; 3 Department of Paediatric Cardiology and Congenital Heart Diseases, German Heart Institute, Berlin, Germany

**Keywords:** CONGENITAL HEART DISEASE, Meta-analysis, aortic valve repair

## Abstract

Aortic valve repair is still emerging, and its role in the treatment of bicuspid aortic valve disease (BAVD) is not yet fully understood. Our objective is to synthesise available evidence on outcomes after surgical aortic valve repair in patients with BAVD. We conducted a systematic review of clinical studies using prespecified methods for searching, identifying and selecting eligible studies in 4 databases, and synthesising results (PROSPERO 2014:CRD42014014415). 2 researchers independently reviewed full-text articles and extracted data. The results of included studies were quantitatively synthesised in frequentist meta-analyses. We included 11 aortic valve repair studies or study arms with a total of 2010 participants. Pooled estimates for the proportion of patients surviving at 30 days, 1 year, 5 years and 10 years were 0.995 (95% CI 0.991 to 0.995), 0.994 (0.989 to 0.999), 0.945 (0.898 to 0.993) and 0.912 (0.845 to 0.979), respectively. The pooled proportion of late deaths from valve-related causes was 0.008 (0.000 to 0.019) at a mean follow-up of 3.5 years. Proportion of patients with valve-related reinterventions was 0.075 (0.037 to 0.113) at a mean follow-up of 3.9 years, and the linearised reintervention rate was 1.3 (0.7 to 1.9) per 100 patient-years. Outcome reporting was insufficient to pool the results for a number of predefined outcomes. In conclusion, existing evidence on aortic valve repair in BAVD is limited to mostly small case series, case–control and small retrospective cohort studies. Despite the low quality, available evidence suggests favourable survival outcomes after aortic valve repair in selected patients with BAVD. Valve-related reinterventions at follow-up are common in all patients undergoing repair surgery.

## Introduction

Bicuspid aortic valve disease (BAVD) is the most common congenital heart disease, affecting 1–2% of the population.^1^ Complications associated with BAVD include aortic stenosis, regurgitation, infective endocarditis and aortic dissection.^2^ Depending on the manifestation of BAVD, different treatment options exist. The most common treatment, surgical replacement of the dysfunctional native valve, has some limitations. Replacement with a mechanical prosthesis requires lifelong anticoagulation medication, potentially constraining the patient's lifestyle. In addition, the choice of the valve size is challenging when patients are still growing. Biological prostheses have not yet proven to be a durable alternative, particularly in younger patients. The Ross procedure is associated not only with subsequent dilation of the aortic annulus but also with an increased risk for aortic insufficiency and pulmonary homograft insufficiency.^3^

In patients with suitable morphology of the diseased valve (typically only patients with aortic regurgitation), repair of the aortic valve is a desirable option. The development of aortic valve repair as an alternative to replacement has been driven by potential benefits of preserving the native valve, which include avoiding anticoagulation medications and fewer complications of the operated valve.^4^ ^5^ However, evidence published to date has been limited to relatively small case series. Previous systematic reviews on valve repair provided little information on the efficacy and safety of this intervention in patients with BAVD.^6^ ^7^ In recent years, several world-leading centres have published their growing experience with aortic valve repair with large sample sizes. Including this newly available information, our aim in this study was to synthesise all available evidence on immediate and long-term outcomes after aortic valve repair in patients with BAVD.

## Methods

At the outset, we developed and made publically available a review protocol with prespecified inclusion and exclusion criteria, relevant outcomes and strategy for statistical analysis on the PROSPERO website of the University of York Centre for Reviews and Dissemination (PROSPERO 2014:CRD42014014415; http://bit.ly/20q683G). Our primary objective was to systematically collect and synthesise available evidence on the effectiveness of aortic valve repair. The protocol contained search strategies for aortic valve repair and replacement studies to allow quantitative assessment of the comparative effectiveness of the two interventions. We found that patients undergoing the two interventions were not comparable, particularly with respect to valve pathology (stenosis vs regurgitation), and therefore we only report the findings of aortic valve repair studies. Table 1 shows the main parameters of this systematic review.

**Table 1:** PICOS table

PICOS	
**P**atient population	▸ Patients with bicuspid aortic valve disease above 1 month of age
**I**nterventions	▸ Surgical valve repair
**C**omparators	▸ Any comparator
**O**utcomes	Complications before discharge: ▸ Reoperation during index admission ▸ Neurologic event Mortality: ▸ 30-day survival ▸ Survival at 1, 5 and 10 years follow-up ▸ Valve-related late mortality at follow-up Complications at follow-up: ▸ Operated valve endocarditis ▸ Thrombosis, embolism and bleeding Reinterventions at follow-up: ▸ Reinterventions on operated valve at follow-up ▸ Freedom from reintervention at 1, 5 and 10 years follow-up
**S**tudy designs	▸ Any study design

### Literature search strategy

We searched online databases MEDLINE (via PubMed; January 1990–October 2014), CINAHL Plus (January 1990–October 2014), EMBASE (January 1990–October 2014) and the Cochrane Library (January 1990–October 2014) using prespecified search terms and phrases (search terms available in the online supplementary material). Database searches were supplemented by the reference lists obtained from three review articles and one clinical practice guideline.^6–9^ We conducted final database searches on 30 October 2014.

10.1136/annrheumdis-2016-210131.supp1 supplementary material

### Eligibility criteria

According to our prespecified inclusion criteria, studies or study arms were eligible for inclusion if they had at least 50 patients undergoing bicuspid aortic valve (BAV) repair; were written in English language and were published in peer-reviewed journals since 1990. Preliminary searches had revealed a paucity of controlled studies, and we therefore did not restrict inclusion to specific study designs. We included studies reporting on patients with BAVD undergoing any form of surgical aortic valve repair, including valve-sparing replacement of the ascending aorta (Yacoub and David procedures). While valve-sparing procedures on the aortic root do not necessarily include reconstruction of the aortic valve, these procedures are often performed concurrently and are aimed at preserving the native valve.

We excluded single case reports, conference abstracts, review articles, references that reported none of the prespecified outcomes and animal studies.

Studies were screened at the abstract and study title level by one researcher (MS). Full texts for articles deemed eligible at this level were retrieved, and references describing the same study were matched and duplicates removed.

Full-text articles were independently assessed for inclusion eligibility by two researchers (MS and HN). Deviating decisions on inclusion were resolved by discussion and consensus between the two researchers.

### Data extraction and critical appraisal

Two researchers (MS and SP) carefully re-examined the included studies and independently extracted prespecified data using a standardised spreadsheet. Selection of outcomes was based on guidelines.^10^ Extracted outcomes were categorised into mortality (30-day; 1-year, 5-year and 10-year and valve-related mortality); complications before discharge (reoperations and neurologic events); complications at follow-up (operated valve endocarditis; and thrombosis, embolism or bleeding event) and reinterventions at follow-up (table 1). From each study, we extracted relevant baseline characteristics and outcomes for all patients who underwent aortic valve repair. Data were extracted as the number of patients with any given outcome. For 1-year, 5-year and 10-year survival, as well as 1-year, 5-year and 10-year reintervention-free survival, we extracted Kaplan-Meier estimates rather than the actual number of patients surviving.

We did not formally assess the risk of bias in included studies. Available instruments, such as the Cochrane risk of bias tool,^11^ have been developed for controlled trials or observational cohort studies. Studies included in this review were primarily case series and typical items included in available risk of bias tools and checklists are therefore not applicable.

### Statistical analysis

To assess between-study heterogeneity, we plotted key patient baseline characteristics against results of key outcomes. We visually explored whether baseline characteristics (including mean patient age; proportion of patients with concomitant ascending aortic procedure; proportion of patients in NYHA class III or IV; proportion of patients with aortic stenosis or regurgitation) were systematically correlated with favourable or unfavourable results. Statistical heterogeneity of study results was quantitatively assessed using the I^2^ statistic.^12^ ^13^ Results of individual studies were pooled using a fixed-effect model when between-study heterogeneity was low (I^2^<25%), and a random-effects model when between-study heterogeneity was moderate to high (I^2^≥25%). In cases where a study had an event proportion of 0 or 1, we imputed the average of the variances of the other studies to obtain an estimate of the variance.^14^

Pooled results are reported as proportions and 95% CIs. Outcomes at follow-up are also presented as number of events per 100 patient-years of follow-up with corresponding 95% CIs. The linearised event rate for each study was calculated as (events/(sample size×mean follow-up time))×100.

Meta-analyses for all outcomes were carried out using the ‘metan’ command in STATA, V.13 (College Station, Texas, USA).

## Results

**Figure 1:**
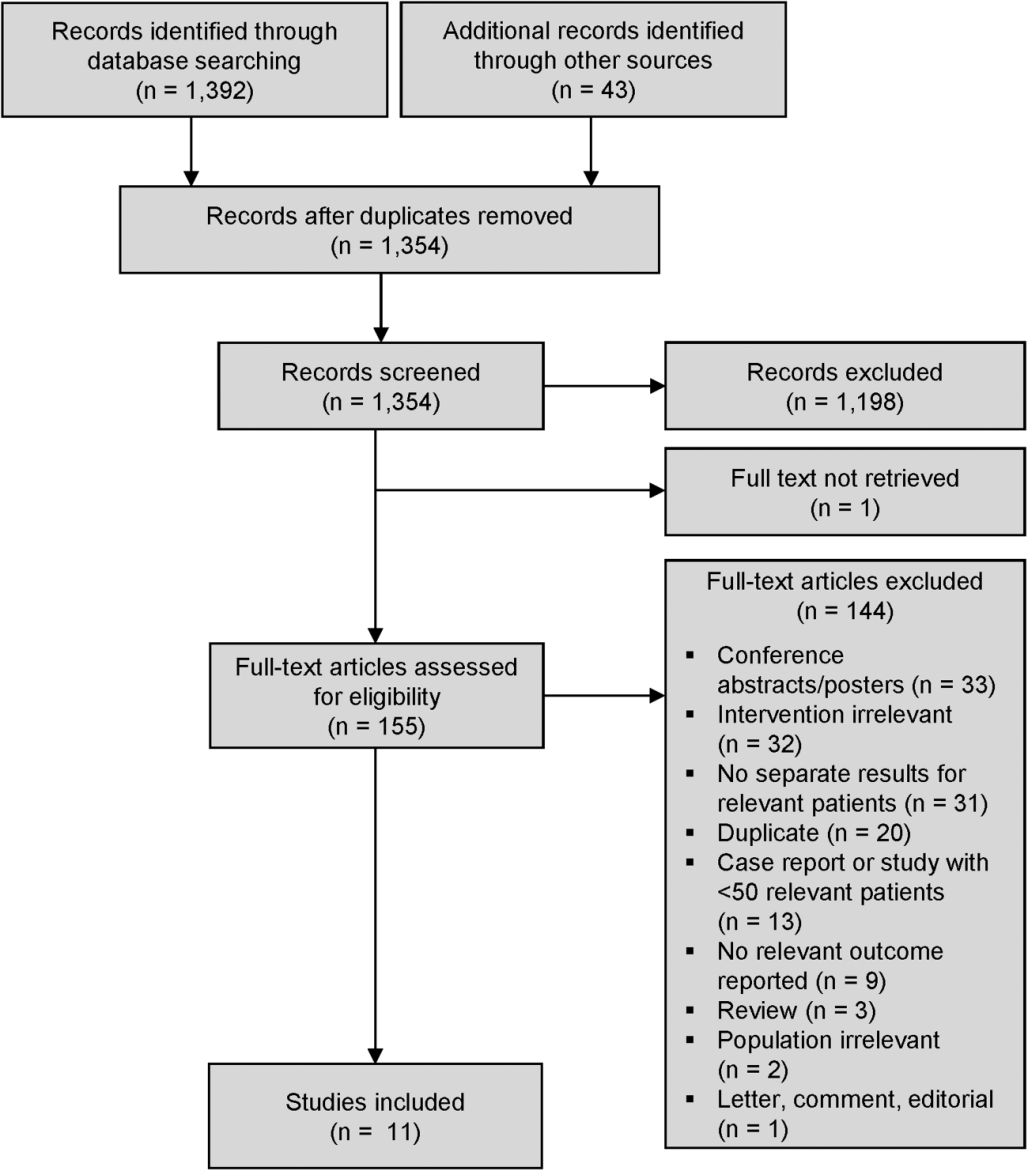
PRISMA flow chart.

Through database searches and reference lists of key reviews and clinical practice guidelines, we identified 1435 references. After eliminating duplicates, 1198 records were excluded at the title and abstract screening stage. Of 155 references assessed at the full-text stage, 11 studies^15–25^ with 2010 patients were deemed eligible for inclusion (figure 1). Details of included studies are provided in the online supplementary material.

### Baseline demographics

**Figure 2:**
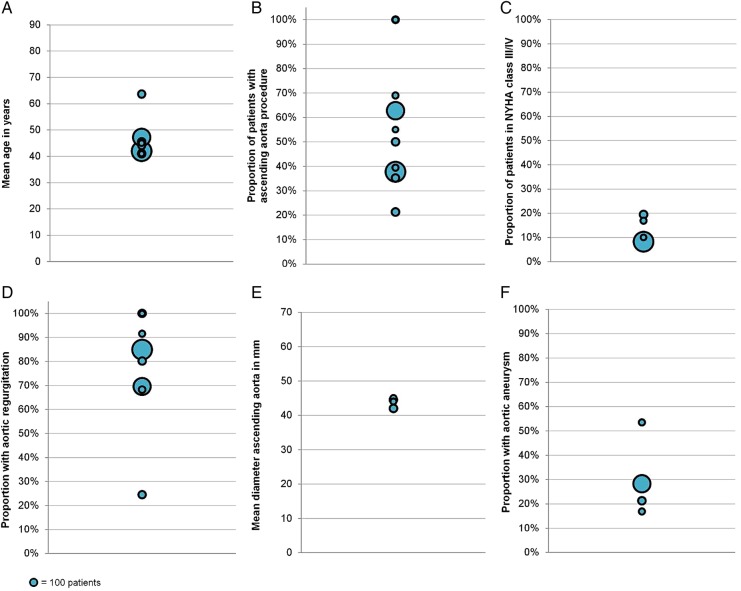
Distribution of patient baseline characteristics among included studies. Each bubble represents one study, with bubble size representing study sample size. (A) Distribution of mean age in years; (B) distribution of proportion of patients with concomitant ascending aorta repair or replacement; (C) distribution of proportion of patients in NYHA class III/IV; (D) distribution of patients with aortic regurgitation; (E) distribution of mean ascending aorta diameter in mm and (F) distribution of patients with aortic aneurysm.

Overall, patient baseline characteristics (table 2) showed relatively little variation across included studies (figure 2). The mean patient age ranged from 41 to 64 years, with an overall patient mean age of 45.3 years across all included studies. Within individual studies, however, age was more varied, with one study including patients ranging from 3 to 86 years^15^ and others including patients between 20 and 68 years of age.^20^ ^21^ The proportion of patients with aortic regurgitation was between 70% and 100% in all studies except for one.^23^ Stenotic valves were present in 12% of patients overall. Of seven studies reporting data for this patient characteristic, five did not include any patients with stenotic valves.

**Table 2:** Patient baseline characteristics

Characteristic		Studies reporting variable (participants)
Patients overall (n)	2010	
Mean age (years)	45.3	11 (2010)
Sex		
Male (%)	82.1%	10 (1956)
Female (%)	17.9%	10 (1956)
Aortic valve pathology		
Aortic regurgitation (%)	81.9%	10 (1956)
Aortic stenosis (%)	12.0%	7 (1241)
Patients in NYHA class III or IV (%)	13.7%	4 (967)
Diameter asc. aorta (mean mm (SD))	43.9 (7.5)	4 (351)
Patients with aortic aneurysm (%)	30.0%	4 (809)
Patients with acute aortic dissection (%)	0.5%	4 (806)
Patients with connective tissue disorder (%)	3.4%	4 (282)
Procedure		
Isolated valve repair (%)	39.5%	6 (1020)
Concomitant asc. aorta repair or replacement (%)	57.1%	10 (1956)

At baseline, ∼30% of patients had aortic aneurysms. This patient characteristic was reported in only four studies, three of which had a prevalence of aortic aneurysm below 30%.

There was some variation in the proportion of patients undergoing concomitant ascending aortic procedures at baseline. Several techniques were used for interventions on the ascending aorta, including variants of annuloplasty, aortoplasty and valve-sparing root replacement (remodelling and reimplantation).

**Figure 3:**
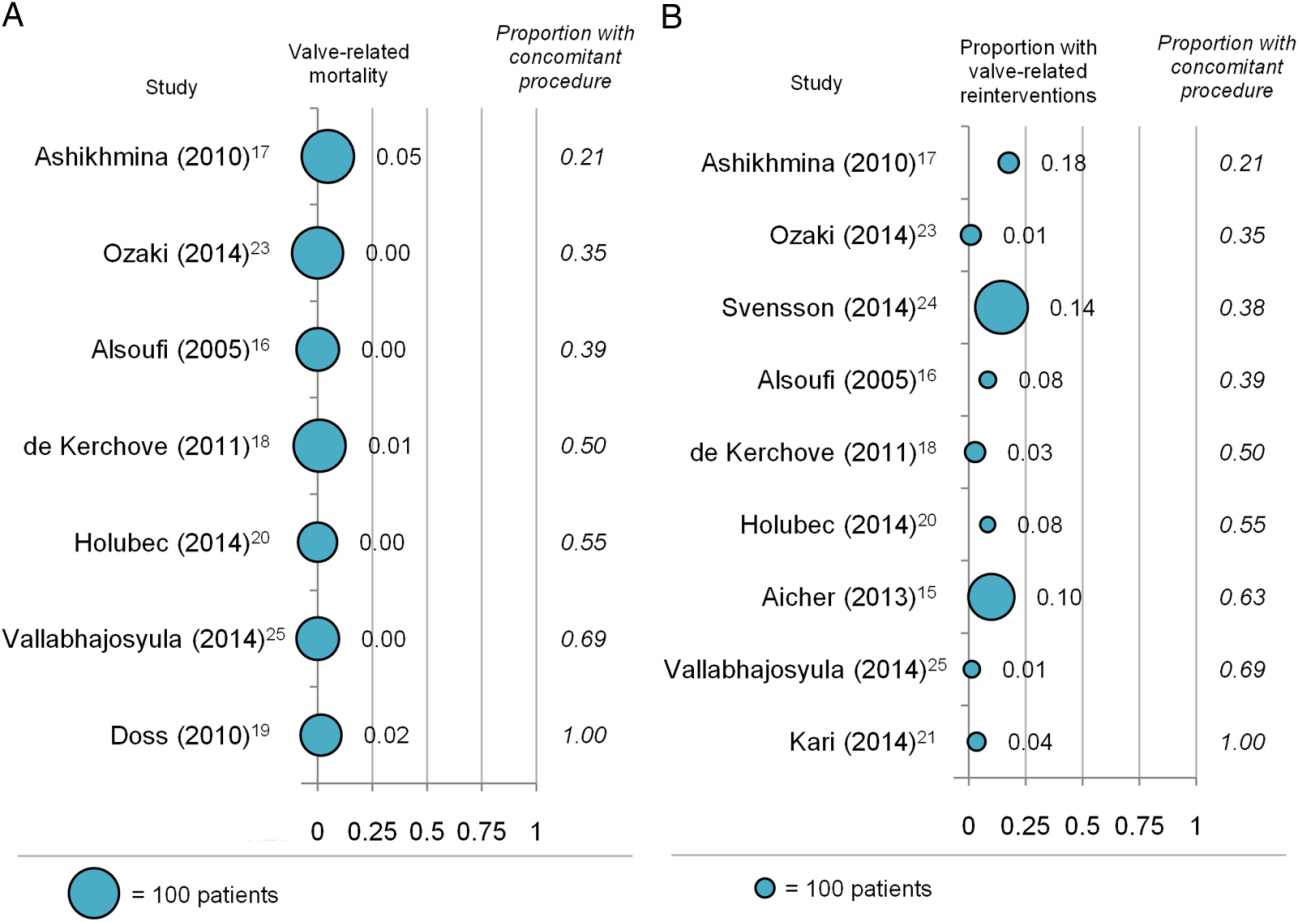
Effect of concomitant ascending aorta procedure on outcomes at follow-up. Each bubble represents one study, with bubble size representing study sample size. Only studies with data for proportion of patients with valve-related deaths at follow-up (A) and valve-related reinterventions at follow-up (B) and the proportion of patients with ascending aorta replacement or repair at the time of valve repair are shown. (A) Results for the proportion of patients with valve-related late mortality. Studies are ranked by ascending proportion of patients with concomitant ascending aorta procedure; (B) results for the proportion of patients with valve-related reinterventions at follow-up. Studies are ranked by ascending proportion of patients with concomitant ascending aorta procedure.

We observed statistical heterogeneity between individual studies for most outcomes. However, we did not detect a systematic relationship between baseline patient characteristics and key outcomes (30-day survival; valve-related mortality; valve-related reinterventions) through visual inspection. For example, as shown in figure 3, valve-related late mortality (Panel A) and valve-related reintervention rates (Panel B) did not systematically vary with the proportion of patients with concomitant ascending aortic procedures. In a similar fashion, no systematic association emerged when we plotted other patient baseline characteristics against these key outcomes (plots presented in online supplementary material).

### Pooled estimates of immediate and follow-up outcomes

Pooled estimates for all outcomes are shown in table 3.

Complications before discharge were not commonly reported. Synthesising the results of four studies (976 patients), we obtained a pooled estimate for the proportion of patients with neurologic events before discharge of 0.007 (95% CI from 0.995 to 0.999 (see table 3); I^2^=28.5%). Reoperations during the initial admission were reported in five studies (422 patients), with a pooled estimated proportion of 0.054 (95% CI 0.010 to 0.099; I^2^=75.2%).

**Table 3:** Pooled results

Outcome	Pooled estimate (95% CI)	Number of studies reporting outcome (participants)
Complications before discharge		
Reoperation during index admission, proportion	0.054 (0.010 to 0.099)*	5 (422),^16–19^ ^25^
Neurologic event, proportion	0.007 (0.000 to 0.018)*	4 (976)^16^ ^18^ ^24^ ^25^
Mortality		
30-day survival, proportion	0.995 (0.991 to 0.999)	9 (1844)^15–20^ ^23–25^
Survival at 1 year, proportion	0.994 (0.989 to 0.999)	5 (1038)^16^ ^17^ ^20^ ^24^ ^25^
Survival at 5 years, proportion	0.945 (0.898 to 0.993)*	4 (1009)^17^ ^23–25^
Survival at 10 years, proportion	0.912 (0.845 to 0.979)*	2 (836)^17^ ^24^
Valve-related late mortality at follow-up		
Proportion	0.008 (0.000 to 0.019)	7 (584)^16–20^ ^23^ ^25^
Per 100 patient-years	0.2 (0.0 to 0.4)	7 (584)^16–20^ ^23^ ^25^
Complications at follow-up		
Operated valve endocarditis		
Proportion	0.011 (0.002 to 0.020)	8 (615)^16^ ^18–23^ ^25^
Per 100 patient-years	0.3 (0.0 to 0.6)	8 (615)^16^ ^18–23^ ^25^
Thrombosis, embolism, and bleeding		
Proportion	0†	4 (304)^16^ ^20^ ^23^ ^25^
Per 100 patient-years	0†	4 (304)^16^ ^20^ ^23^ ^25^
Reinterventions at follow-up		
Reinterventions on operated valve at follow-up		
Proportion	0.075 (0.037 to 0.113)*	10 (1944)^15–18^ ^20–25^
Per 100 patient-years	1.3 (0.7 to 1.9)*	9 (1385)^16–18^ ^20–25^
Freedom from reintervention at 1 year	0.952 (0.938 to 0.967)	2 (799)^16^ ^24^
Freedom from reintervention at 5 years	0.934 (0.874 to 0.993)*	5 (1026)^16^ ^22–25^
Freedom from reintervention at 10 years	0.800 (0.760 to 0.839)*	2 (1287)^15^ ^24^

The table shows the number of studies reporting each outcome and the corresponding number of participants in these studies.

*Weights from random effects analysis. Results without indicator are from fixed-effect analysis with inverse variance weighting.

†No CIs computed because of 0 variance in all four studies reporting the outcome.

**Figure 4:**
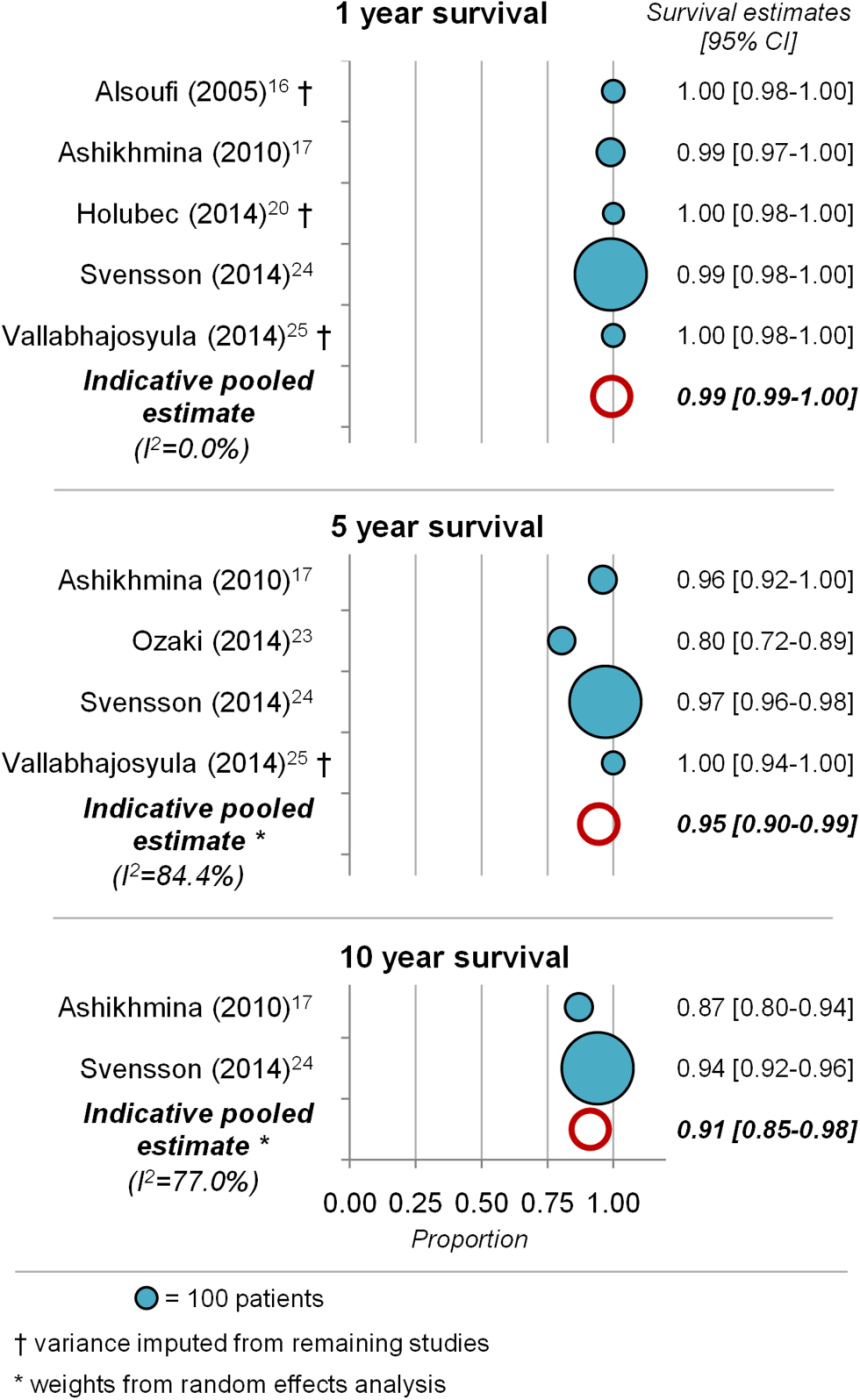
Survival at follow-up. Kaplan-Meier estimates from included studies for survival at 1, 5 and 10 years follow-up. Each bubble represents one study, with bubble size representing study sample size. Pooled estimates of survival are shown as empty circles.

Survival within 30 days of aortic valve repair was 0.995 (95% CI 0.991 to 0.999; I^2^=0.0%). Figure 4 shows the Kaplan-Meier estimates of survival at 1, 5 and 10 years from studies reporting these estimates along with pooled survival estimates. Pooled estimates showed a decrease in survival from 0.994 (95% CI 0.989 to 0.999; I^2^=0.0%) at 1 year and 0.945 (95% CI 0.898 to 0.993; I^2^=84.4%) at 5 years to 0.912 (95% CI 0.845 to 0.979; I^2^=77.0%) at 10 years. Survival at 10 years of follow-up was extracted from only two studies with estimates of 87%^17^ and 94%,^24^ respectively.

There was less variation in the proportion of patients with valve-related deaths at follow-up. We obtained a pooled estimate of 0.008 (95% CI 0.000 to 0.019; I^2^=0.0%) for this outcome from seven studies with mean follow-up ranging from 2 to 5.1 years (mean 3.5 years). Pooled linearised valve-related mortality was 0.2 per 100 patient-years (95% CI 0.0 to 0.4; I^2^=0.0%).

**Figure 5:**
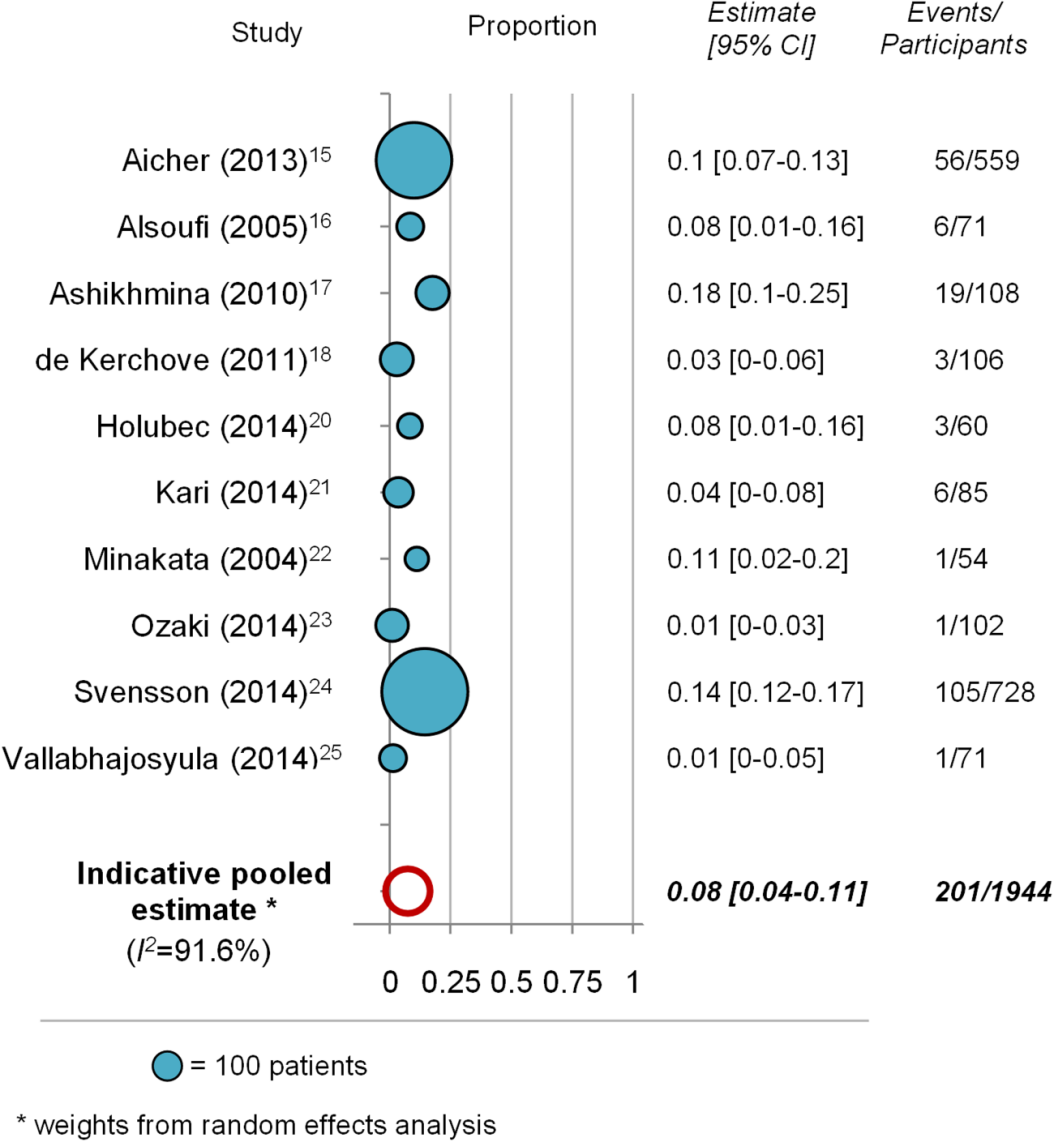
Proportion of patients with valve-related reinterventions at follow-up. Each bubble represents one study, with bubble size representing study sample size. Pooled estimate of the proportion is shown as empty circle.

Proportion of patients with valve-related reinterventions at follow-up ranged from 0.01 to 0.18 in individual studies; the pooled estimate was 0.075 (95% CI 0.037 to 0.113; I^2^=91.6%; figure 5). The mean follow-up time in ten studies reporting the outcome ranged from 2 to 9 years (mean 3.9 years). Pooled estimate for the linearised reintervention rate was 1.3 per 100 patient-years (95% CI 0.7 to 1.9; I^2^=69.2%).

Freedom from reintervention as measured by extracted Kaplan-Meier estimates decreased from a pooled proportion of 0.952 (95% CI 0.938 to 0.967; I^2^=0.0%) at 1 year and 0.934 (95% CI 0.874 to 0.993; I^2^=94.1%) at 5 years to 0.800 (95% CI 0.760 to 0.839; I^2^=68.8%) at 10 years follow-up. Only two studies contributed to the pooled estimate of reintervention-free survival at 1 year (Kaplan-Meier estimates: 96.8%^16^ and 95.0%^24^). Reintervention-free survival at 10 years was also only reported in two studies (Kaplan-Meier estimates: 82.0%^15^ and 78.0%^24^).

At a study-level mean follow-up time of 3.3 years (mean follow-up ranging from 2.0 to 5.1 years across studies), endocarditis of the operated valve was observed in an estimated 1.1% of patients (95% CI 0.002 to 0.020; I^2^=0.0%). The pooled linearised rate for operated valve endocarditis was 0.3 per 100 patient-years (95% CI 0.0 to 0.6; I^2^=0.0%). Other complications at follow-up, captured in the composite indicator for thrombosis, embolism and bleeding, did not occur in the four studies reporting these outcomes.

## Discussion

In this paper, we systematically assessed and synthesised the available evidence on outcomes after aortic valve repair in patients with BAVD. In this comprehensive assessment, which included all techniques preserving the native valve, we found 11 studies matching our inclusion criteria. The evidence base consisted of single-centre case series with mean patient age at the study-level between 41 and 64 years, some of which retrospectively compared results between different types of valve repair. Pooled estimates from our meta-analyses suggest favourable 30-day and long-term survival after BAV repair. Although the durability of aortic valve repair in this patient population remains uncertain, our meta-analysis suggests that this intervention can contribute to a positive outlook for patients diagnosed with BAVD, who, as observed in a population-based study in Minnesota, can expect similar 25-year survival as the general population once the diagnosis is made.^26^ Aortic valve-preserving surgery is still undergoing changes as a surgical technique, and its use is limited to centres of excellence with experienced surgeons.^27^ The favourable findings of our systematic review should be interpreted against this background.

Anatomic features of the individual patient's aorta (in particular dilated aortic root) and configuration of the diseased valve are important considerations when choosing the type of valve repair. At the study level, we did not detect a systematic association between concomitant ascending aorta procedures and key outcomes. Two of the included studies did not find a statistically significant difference in survival at follow-up between patients undergoing isolated valve repair and patients with concomitant aortic root replacement or sinotubular junction remodelling.^15^ ^25^ Other studies found a positive effect from aortic root replacement or remodelling of the sinotubular junction at the time of aortic valve repair;^15^ ^16^ ^18^ this finding has led to increased advocacy for aggressive root replacement even in patients with mild or moderate root dilation.^18^

Our pooled estimate of 30-day survival after aortic valve repair was 0.995 (95% CI 0.991 to 0.999). This suggests a considerably lower early mortality rate compared to a previous meta-analysis which did not focus exclusively on patients with BAVD (pooled estimated early mortality 0.026, 95% CI 0.014 to 0.044),^6^ potentially highlighting differences and the need for differentiations of patients with bicuspid versus tricuspid valves. However, two studies found no difference in long-term survival between the two valve types,^20^ ^22^ although this might have been due to the rare occurrence of late deaths after the procedure. Our results confirm and extend the findings of another systematic review of 30, often small, aortic valve repair studies in BAVD patients which found a median 30-day mortality rate of 0%.^7^

Reinterventions at follow-up are not uncommon after BAV repair. Pooled estimates for freedom from valve-related reinterventions at follow-up showed a decline from 93.4% at 5 years to 80.0% at 10 years. This suggests increased failure of valve repair after 10 years. In the two studies reporting reintervention-free survival at 10 years, reoperation was performed mainly for recurrent regurgitation^15^ and cusp prolapse.^24^

Whether replacement of the valve is a more durable alternative remains elusive. Some series of aortic valve replacement in BAVD patients report proportions of patients with reinterventions well below 5%^28^ ^29^ while others report considerably higher rates.^30^ ^31^ However, a direct comparison of outcomes after valve repair and replacement may not be feasible. Repair techniques are not practical for all aortic valve pathologies. We found that most series excluded patients with aortic valve stenosis from aortic valve repair. In addition, expected benefits from preserving the native valve may play a more prominent role in treatment decisions for younger and healthier patients. The absence of lifelong need for anticoagulation medication may be a stronger argument for young patients who want to maintain an active lifestyle. Such differences in the patient groups undergoing aortic valve repair or replacement can systematically influence the results of published case series. Indeed, authors of included studies mentioned that only selected patients underwent repair.^17^ ^18^ ^22^ We included only studies with 50 or more participants to obtain more robust results about the effectiveness of aortic valve repair compared to very small case series. In our sample of 11 studies, none was conducted exclusively in young patients. The low number of patients <30 years seen at any particular centre may prohibit the publication of large patient series focusing on this young patient group. This hints at a gap in knowledge about outcomes after aortic valve repair in the group which is most likely to benefit from it, young adults.

Comparison of surgical aortic valve repair and replacement is only feasible for similar patient groups in the setting of a controlled clinical trial. Our database search retrieved only one such study with sufficiently large sample size which found no statistically significant difference in 10-year survival and freedom from reoperation between aortic valve repair and replacement.^17^

Compared to another systematic review published in 2013,^7^ our study shows less variation and slightly better results reported by individual centres. Different inclusion criteria and a gap in time between the execution of the two reviews can serve as possible explanation for differences in findings. First, we excluded very small studies. Included studies were conducted at larger centres with more experienced surgeons, which is likely to positively impact on desirable outcomes. Second, know-how in surgical repair of bicuspid aortic valves is still evolving, suggesting favourable results in more recently operated patients. In one study comparing the risk of patients for undergoing aortic valve replacement at follow-up between patients who had their initial aortic valve repair before and after 2000, a trend towards better outcomes after 2000 was discovered, suggesting a learning curve effect for operating surgeons.^17^ In addition, it is possible that centres are improving with respect to appropriate patient identification and selection for aortic valve repair. Michelena *et al*^32^ identified gaps in the knowledge about BAVD and maintain that research into this complex disease has ‘generated more questions than answers’.

### Limitations

Our study has several limitations. First, data on specific outcomes were not always available from all included studies, leading to a small evidence base for some outcomes. Second, included studies are mostly case series or retrospective observational studies, which rank the overall evidence between levels 3 and 4.^33^ Bias is more likely to occur in methodologically less rigorous study designs and systematic reviews including such study designs are prone to bias themselves.^34^ Selection of healthier patients for surgical methods that are still in development can bias results towards more beneficial outcomes. Third, despite our best efforts to account for differences in patient baseline characteristics, unmeasured traits could have confounded the results. For example, fusion of the right and left coronary cusps is associated with more aggressive progression of aortic dilation.^35^ However, we were not able to assess whether valve morphology impacted on patients outcomes, as this characteristic was not commonly reported in included studies. Finally, aortic valve repair is not a homogenous surgical technique. To accommodate characteristics of individual patients, a variety of approaches is used, including replacement or repair of the ascending aorta. The objective of this meta-analysis was to synthesise available evidence on aortic-valve preserving surgery in patients with BAVD, and we therefore did not distinguish between specific surgical techniques. The conclusions drawn from this analysis may therefore not be generalisable for specific techniques.

## Conclusions

Our systematic review demonstrated that the clinical literature on outcomes after aortic valve repair in BAVD patients is still limited to mostly case series including in some cases retrospective comparisons of repair techniques within individual centres. Methodologically rigorous controlled studies comparing outcomes after aortic valve repair with alternatives, specifically aortic valve replacement, are needed. Aortic valve repair is still developing at individual centres, and its role in the treatment of BAVD is not yet fully understood. While mainly used in aortic valve insufficiency, additional centre-specific applications for the treatment of stenotic bicuspid valves have been described. Synthesising the available evidence from case series, we found that aortic valve repair in patients with BAVD appears to be associated with favourable survival. No systematic influence of concomitant ascending aorta surgery at the time of valve repair on patient outcome was shown in the included studies. Questions remain regarding the durability of the procedure as valve-related reinterventions at 10 years of follow-up are common in all patients undergoing repair surgery.

## References

[1] Siu SC, Silversides CK Bicuspid aortic valve disease. J Am Coll Cardiol 2010;55:2789–800. 10.1016/j.jacc.2009.12.068 20579534

[2] Ward C Clinical significance of the bicuspid aortic valve. Heart 2000;83:81–5. 10.1136/heart.83.1.81 10618341 PMC1729267

[3] David TE, David C, Woo A The Ross procedure: outcomes at 20 years. J Thorac Cardiovasc Surg 2014;147:85–93. 10.1016/j.jtcvs.2013.08.007 24084276

[4] Aicher D, Schäfers H-J Aortic valve repair—current status, indications, and outcomes. Semin Thorac Cardiovasc Surg 2012;24:195–201. 10.1053/j.semtcvs.2012.08.003 23200074

[5] Boodhwani M, El Khoury G Aortic valve repair. Oper Tech Thorac Cardiovasc Surg 2009;14:266–80. 10.1053/j.optechstcvs.2009.11.002 21092791

[6] Saczkowski R, Malas T, de Kerchove L Systematic review of aortic valve preservation and repair. Ann Cardiothorac Surg 2013;2:3–9. 10.3978/j.issn.2225-319X.2013.01.07 23977553 PMC3741817

[7] Vohra HA, Whistance RN, De Kerchove L Valve-preserving surgery on the bicuspid aortic valve. Eur J Cardiothorac Surg 2013;43:888–98. 10.1093/ejcts/ezs664 23293321

[8] Tourmousoglou C, Lalos S, Dougenis D Is aortic valve repair or replacement with a bioprosthetic valve the best option for a patient with severe aortic regurgitation? Interact Cardiovasc Thorac Surg 2014;18:211–18. 10.1093/icvts/ivt453 24203980 PMC3895061

[9] Nishimura RA, Otto CM, Bonow RO, American College of Cardiology/American Heart Association Task Force on Practice Guidelines. 2014 AHA/ACC guideline for the management of patients with valvular heart disease: a report of the American College of Cardiology/American Heart Association Task Force on Practice Guidelines. J Am Coll Cardiol 2014;63:e57–185. 10.1016/j.jacc.2014.02.536 24603191

[10] Akins CW, Miller DC, Turina MI Guidelines for reporting mortality and morbidity after cardiac valve interventions. Ann Thorac Surg 2008;85:1490–5. 10.1016/j.athoracsur.2007.12.082 18355567

[11] Higgins JP, Altman DG, Gøtzsche PC, Cochrane Bias Methods Group; Cochrane Statistical Methods Group. The Cochrane Collaboration's tool for assessing risk of bias in randomised trials. BMJ 2011;343:d5928 10.1136/bmj.d5928 22008217 PMC3196245

[12] Higgins JP, Thompson SG Quantifying heterogeneity in a meta-analysis. Stat Med 2002;21:1539–58. 10.1002/sim.1186 12111919

[13] Higgins JP, Thompson SG, Deeks JJ Measuring inconsistency in meta-analyses. BMJ 2003;327:557–60. 10.1136/bmj.327.7414.557 12958120 PMC192859

[14] Tabaee A, Anand VK, Barrón Y Endoscopic pituitary surgery: a systematic review and meta-analysis. J Neurosurg 2009;111:545–54. 10.3171/2007.12.17635 19199461

[15] Aicher D, Schneider U, Schmied W Early results with annular support in reconstruction of the bicuspid aortic valve. J Thorac Cardiovasc Surg 2013;145:S30–4. 10.1016/j.jtcvs.2012.11.059 23260458

[16] Alsoufi B, Borger MA, Armstrong S Results of valve preservation and repair for bicuspid aortic valve insufficiency. J Heart Valve Dis 2005;14:752–8. 16359055

[17] Ashikhmina E, Sundt TM III, Dearani JA Repair of the bicuspid aortic valve: a viable alternative to replacement with a bioprosthesis. J Thorac Cardiovasc Surg 2010;139:1395–401. 10.1016/j.jtcvs.2010.02.035 20392456

[18] de Kerchove L, Boodhwani M, Glineur D Valve sparing-root replacement with the reimplantation technique to increase the durability of bicuspid aortic valve repair. J Thorac Cardiovasc Surg 2011;142:1430–8. 10.1016/j.jtcvs.2011.08.021 21955470

[19] Doss M, Risteski P, Sirat S Aortic root stability in bicuspid aortic valve disease: patch augmentation plus reduction aortoplasty versus modified David type repair. Eur J Cardiothorac Surg 2010;38:523–7. 10.1016/j.ejcts.2010.03.006 20456970

[20] Holubec T, Zacek P, Jamaliramin M Valve cuspidity: a risk factor for aortic valve repair? J Card Surg 2014; 29:585–92. 10.1111/jocs.12382 24919866

[21] Kari FA, Kvitting JP, Stephens EH Tirone David procedure for bicuspid aortic valve disease: impact of root geometry and valve type on mid-term outcomes. Interact Cardiovasc Thorac Surg 2014;19:375–81; discussion 381 10.1093/icvts/ivu123 24903440

[22] Minakata K, Schaff HV, Zehr KJ Is repair of aortic valve regurgitation a safe alternative to valve replacement? J Thorac Cardiovasc Surg 2004;127:645–53. 10.1016/j.jtcvs.2003.09.018 15001892

[23] Ozaki S, Kawase I, Yamashita H Reconstruction of bicuspid aortic valve with autologous pericardium—usefulness of tricuspidization. Circ J 2014;78:1144–51. 10.1253/circj.CJ-13-1335 24614492

[24] Svensson LG, Al Kindi AH, Vivacqua A Long-term durability of bicuspid aortic valve repair. Ann Thorac Surg 2014;97:1539–47; discussion 1548 10.1016/j.athoracsur.2013.11.036 24680032

[25] Vallabhajosyula P, Komlo C, Szeto WY Root stabilization of the repaired bicuspid aortic valve: subcommissural annuloplasty versus root reimplantation. Ann Thorac Surg 2014;97:1227–34. 10.1016/j.athoracsur.2013.10.071 24418204

[26] Michelena HI, Khanna AD, Mahoney D Incidence of aortic complications in patients with bicuspid aortic valves. JAMA 2011;306:1104–12. 10.1001/jama.2011.1286 21917581

[27] Bajona P, Feindel CM Bicuspid-aortic valve surgery: repair or replace? Curr Opin Cardiol 2010;25:119–23. 10.1097/HCO.0b013e328335ffde 20104175

[28] Charitos EI, Stierle U, Petersen M The fate of the bicuspid valve aortopathy after aortic valve replacement. Eur J Cardiothorac Surg 2014;45:e128–35. 10.1093/ejcts/ezt666 24482387

[29] Girdauskas E, Disha K, Secknus M Increased risk of late aortic events after isolated aortic valve replacement in patients with bicuspid aortic valve insufficiency versus stenosis. J Cardiovasc Surg (Torino) 2013;54:653–9. 24002396

[30] Borger MA, Preston M, Ivanov J Should the ascending aorta be replaced more frequently in patients with bicuspid aortic valve disease? J Thorac Cardiovasc Surg 2004;128:677–83. 10.1016/j.jtcvs.2004.07.009 15514594

[31] Hanke T, Charitos EI, Stierle U The Ross operation—a feasible and safe option in the setting of a bicuspid aortic valve? Eur J Cardiothorac Surg 2010;38:333–9. 10.1016/j.ejcts.2010.01.064 20359904

[32] Michelena HI, Prakash SK, Della Corte A Bicuspid aortic valve: identifying knowledge gaps and rising to the challenge from the International Bicuspid Aortic Valve Consortium (BAVCon). Circulation 2014;129:2691–704. 10.1161/CIRCULATIONAHA.113.007851 24958752 PMC4145814

[33] OCEBM Levels of Evidence Working Group. The Oxford Levels of Evidence 2. Oxford Centre for Evidence-Based Medicine. http://www.cebm.net/index.aspx?o=5653

[34] Sterne JA, Egger M, Smith GD Systematic reviews in health care: investigating and dealing with publication and other biases in meta-analysis. BMJ 2001;323:101–5. 11451790 10.1136/bmj.323.7304.101 PMC1120714

[35] Ikonomidis JS, Ruddy JM, Benton SM Aortic dilatation with bicuspid aortic valves: cusp fusion correlates to matrix metalloproteinases and inhibitors. Ann Thorac Surg 2012;93:457–63. 10.1016/j.athoracsur.2011.09.057 22206960 PMC3265643

