# Supplementary material for: Bicuspid aortic valve disease: systematic review and meta-analysis of surgical aortic valve repair
Source: Open Heart. 2016 Dec 16;3(2):e000502. doi: 10.1136/openhrt-2016-000502 (PMC5174788; doi:10.1136/openhrt-2016-000502)
Supplement: supplementary material [file openhrt-2016-000502supp.pdf]

SUPPLEMENTAL MATERIAL

BICUSPID AORTIC VALVE DISEASE: SYSTEMATIC REVIEW AND META-ANALYSIS OF SURGICAL AORTIC VALVE REPAIR

Maximilian Salcher [1]*; Huseyin Naci [1]; Sarah Pender [1,2]; Titus Kuehne [3]; CARDIOPROOF [4]; Marcus Kelm [3]

[1] LSE Health, Department of Social Policy, London School of Economics and Political Science, London, United Kingdom;

[2] London School of Hygiene and Tropical Medicine, London, United Kingdom;

[3] Department of Paediatric Cardiology and Congenital Heart Diseases, German Heart Institute, Berlin, Germany.

[4] This work was carried out as part of the CARDIOPROOF project (www.cardioproof.eu), partially funded by the European Commission under FP7. CARDIOPROOF's partners are the following: Edwin Morley-Fletcher (LYNKEUS), Project Coordinator; Titus Kühne (DHZB), Principal Investigator; Anja Hennemuth (FRAUNHOFER MEVIS); David Manset (GNÚBILA); Alistair McGuire (LSE); Gernot Plank (MUG); Olivier Ecabert (SIEMENS AG); Giacomo Pongiglione (OPBG); Vivek Muthurangu (UCL).

* Corresponding Author. LSE Health and Social Care, Cowdray House; London School of Economics and Political Science; Houghton Street, London WC2A 2AE, United Kingdom. Tel: +44 2079556959; Fax: +44 2079556803; Email: m.salcher@lse.ac.uk

Contents

A. Estimates from individual studies and pooled results S3
B. Impact of patient baseline characteristics on outcomes S10
C. Included studies S14
D. Search strategy S18

A. Estimates from individual studies and pooled results

In this section we present summary data for all outcomes in individual studies and pooled estimates including 95% confidence intervals (CI). Figures S1-S8 follow the order of outcomes presented in Table 3 of the main body of the manuscript with the exception of the composite endpoint of thrombosis, embolism, and bleeding event at follow-up for which no pooled estimate was calculated because no event was reported in all four studies reporting the outcome.

Figure S1: Reoperations during index admission

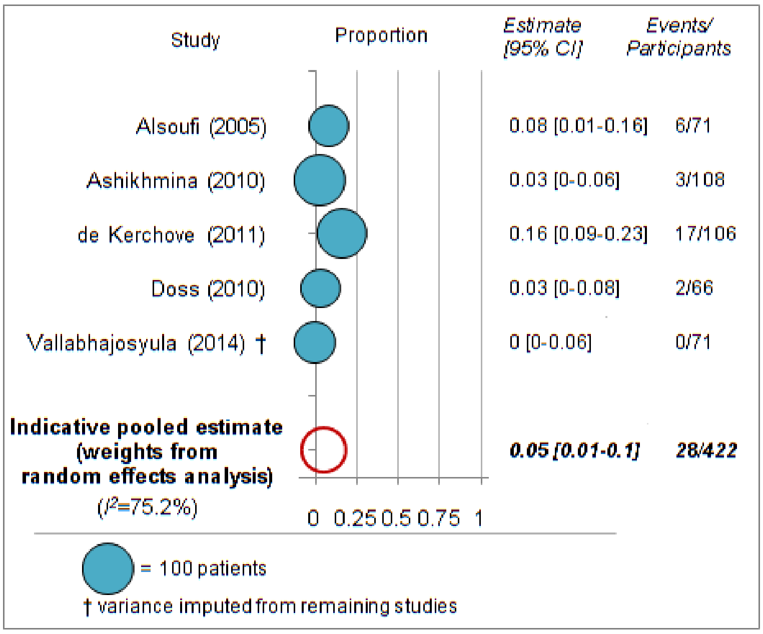

Figure S2: Neurological complications during index admission

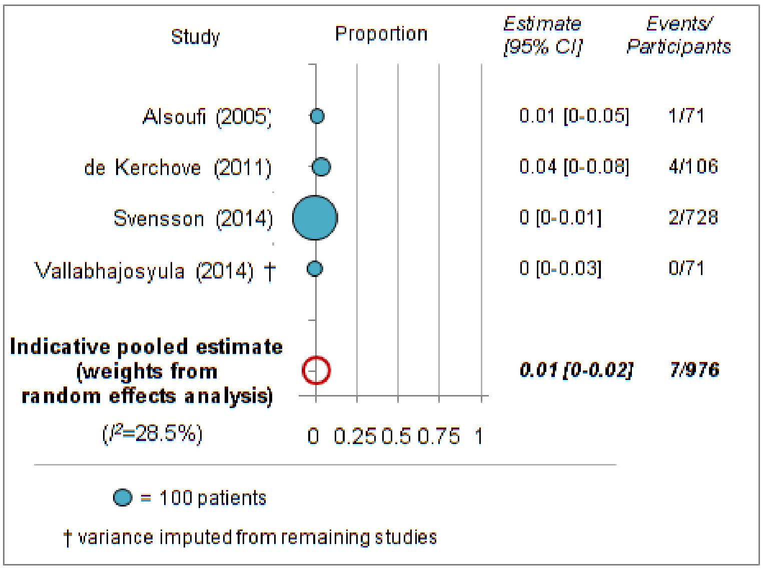

Figure S3: 30-day survival

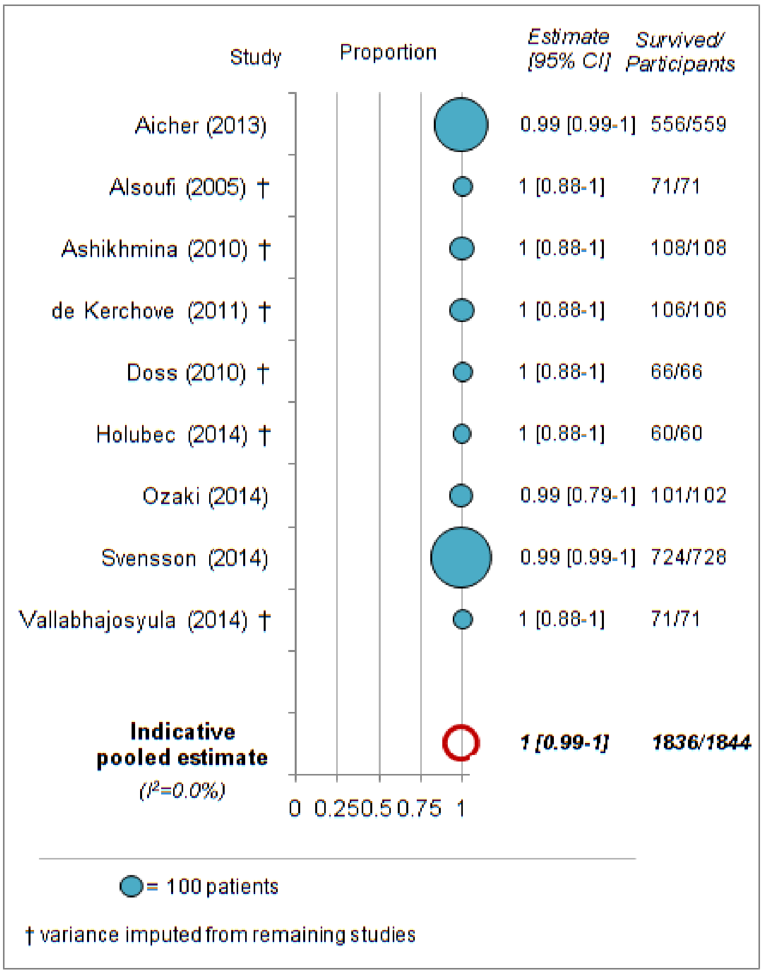

Figure S4: Survival at 1, 5, and 10 years of follow-up

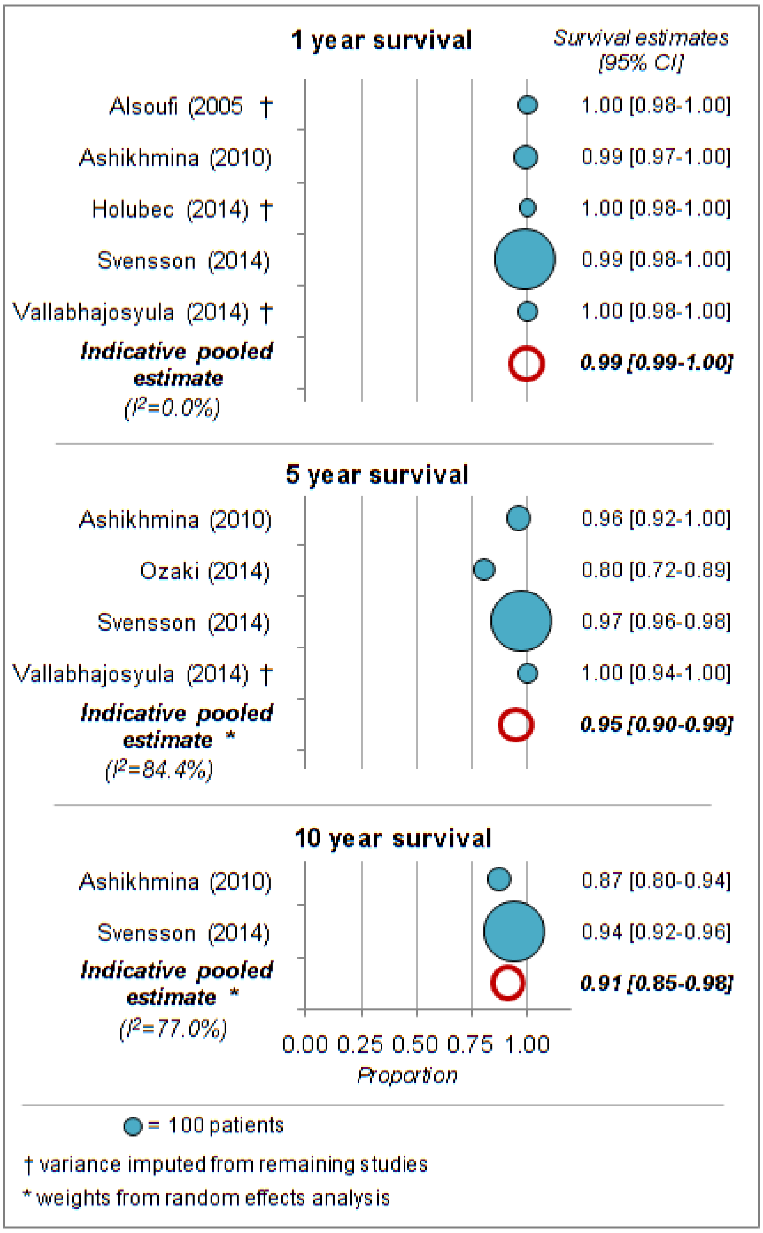

Figure S5: Valve-related mortality at follow-up

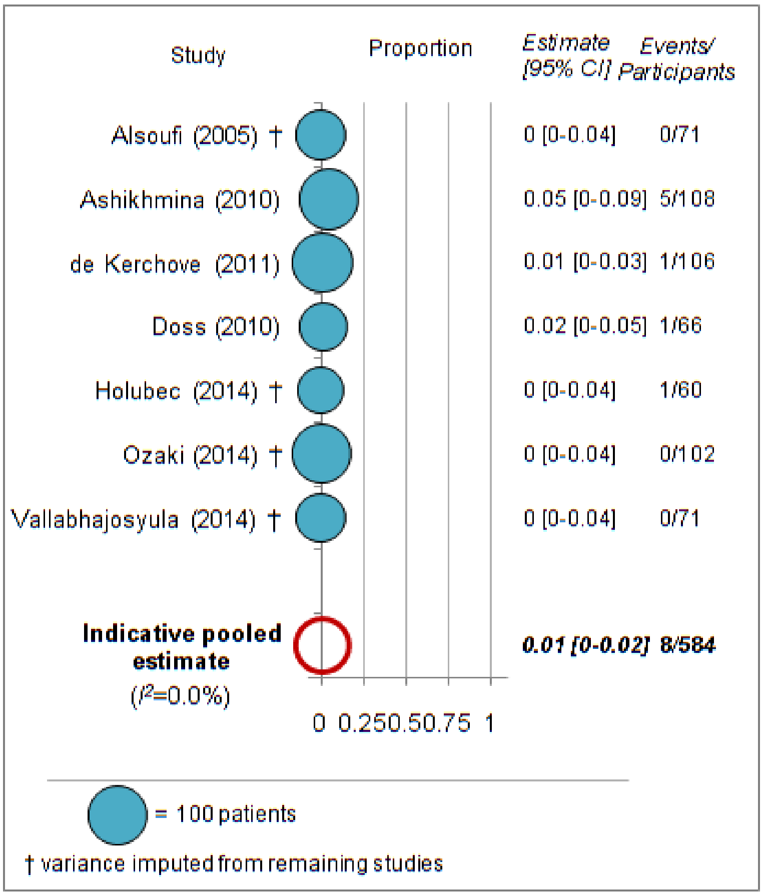

Figure S6: Operated valve endocarditis at follow-up

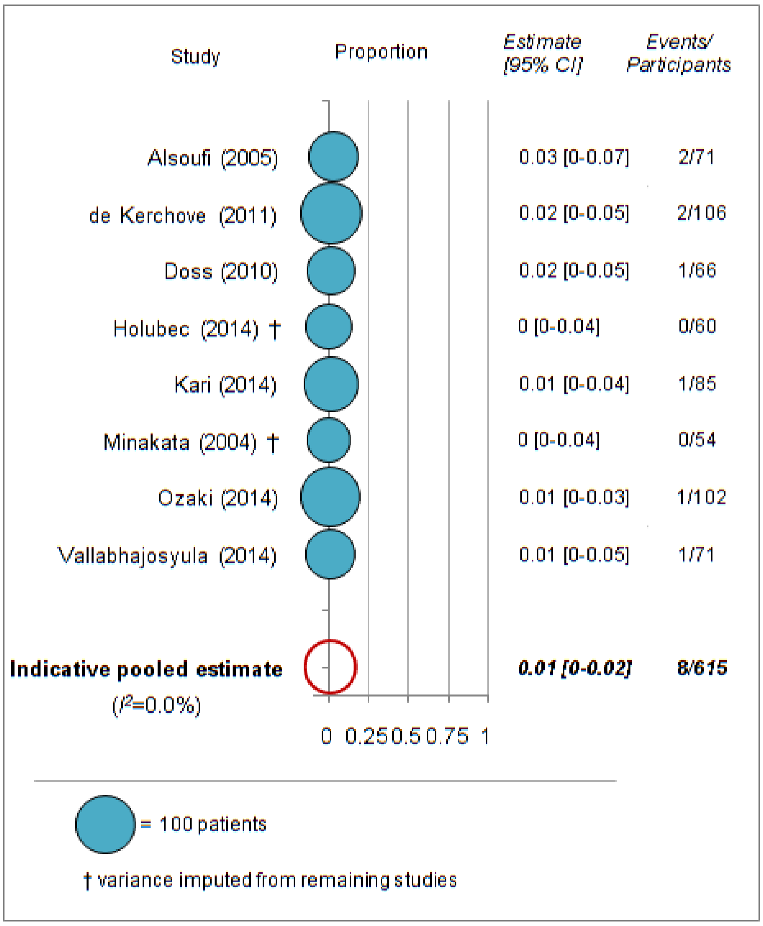

Figure S7: Reinterventions on operated valve at follow-up

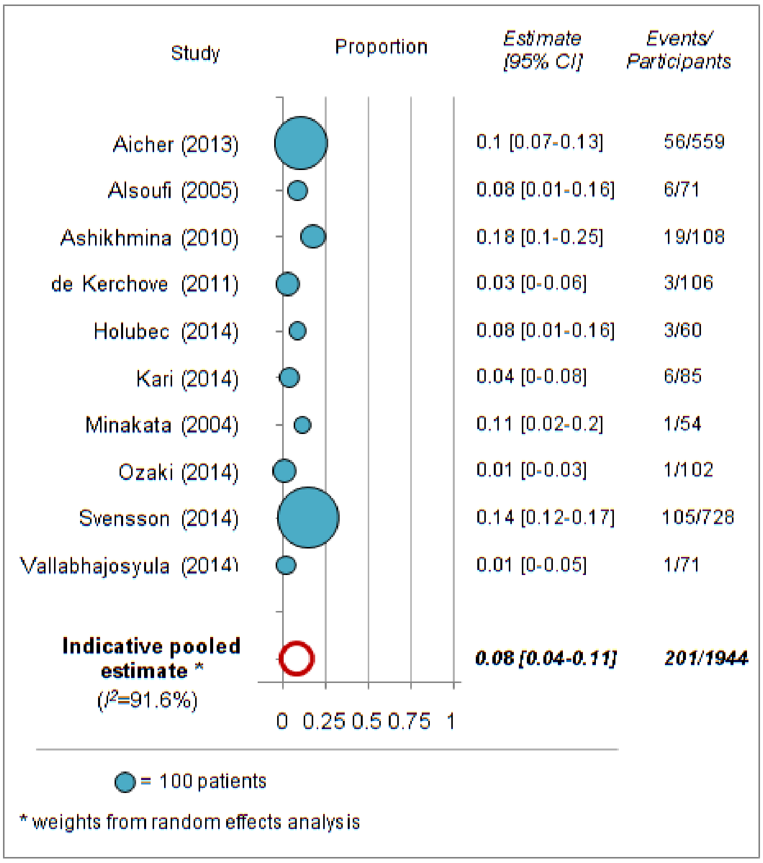

Figure S8: Reintervention-free survival at 1, 5, and 10 years of follow-up

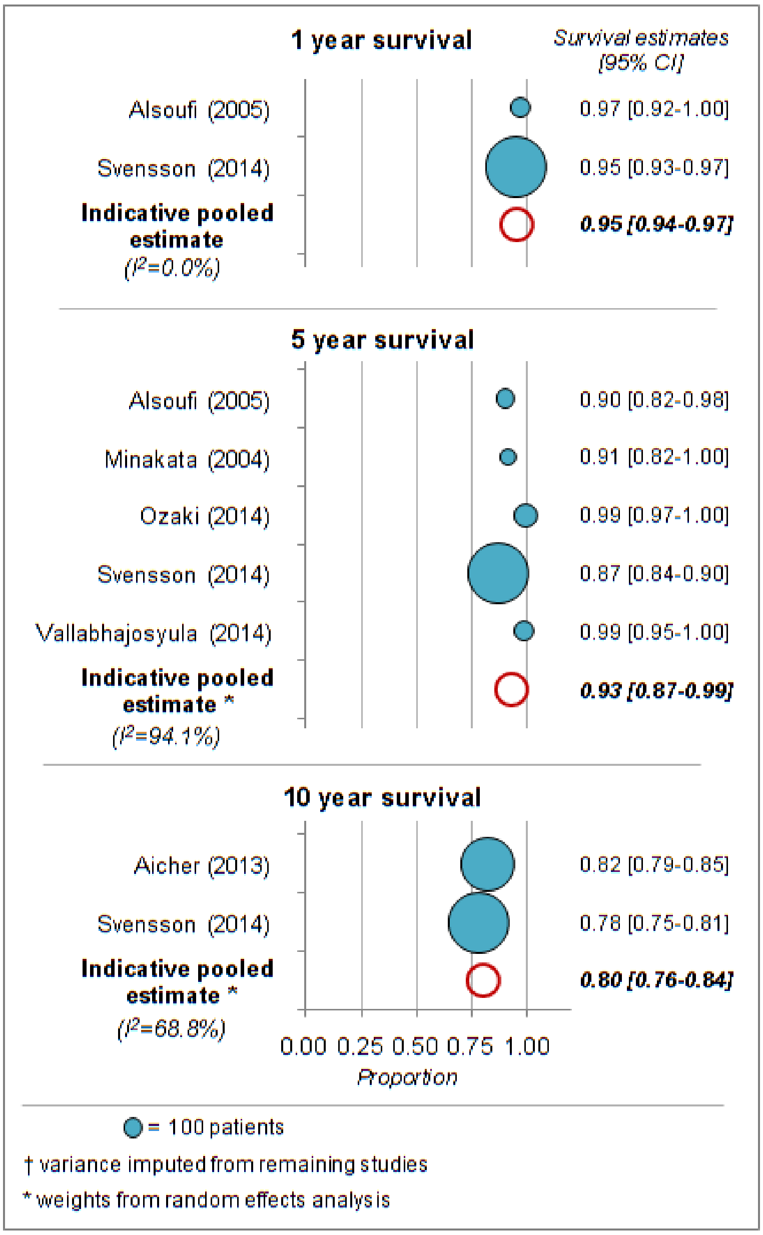

B. Impact of patient baseline characteristics on outcomes

We inspected visually whether key patient baseline characteristics at the study level systematically impacted on three key outcomes (30-day survival; valve-related mortality; valve-related reinterventions).

We assessed the impact of:

- Mean age of patients
- Proportion of patients undergoing concomitant ascending aorta procedures at the same time as valve repair
- Proportion of patients with aortic regurgitation
- Proportion of patients in NYHA classes III or IV

For each outcome, we plotted included studies against these baseline characteristics. Studies were ranked by their corresponding value for the baseline characteristic. For example, for studies reporting 30-day survival rates, we ranked these studies by ascending mean age. We then visually evaluated whether results appeared systematically better or worse in studies with higher mean age.

In each figure (S9-S11), the outcome is plotted against patient mean age (Panel A); proportion of patients with concomitant ascending aorta procedure (Panel B); proportion of patients with aortic regurgitation (Panel C); and proportion of patients in NYHA class III/IV (Panel D).

Overall, we did not detect a discernible systematic impact of these four patient baseline characteristics on key outcomes. There was limited information available for the proportion of patients in NYHA class III/IV.

Figure S9: Effect of study-level baseline patient characteristics on 30-day survival

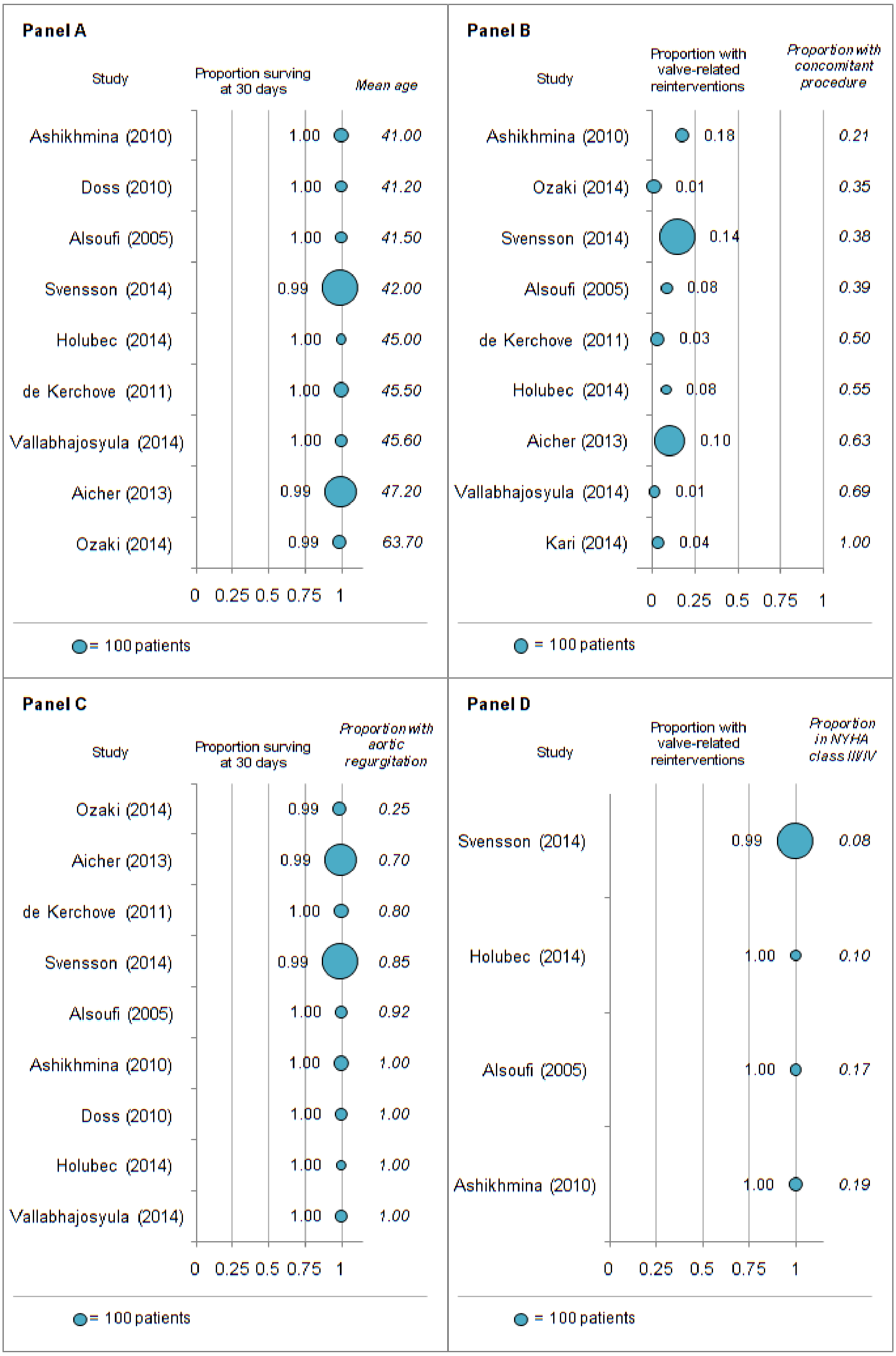

Figure S10: Effect of study-level baseline patient characteristics on valve-related late mortality

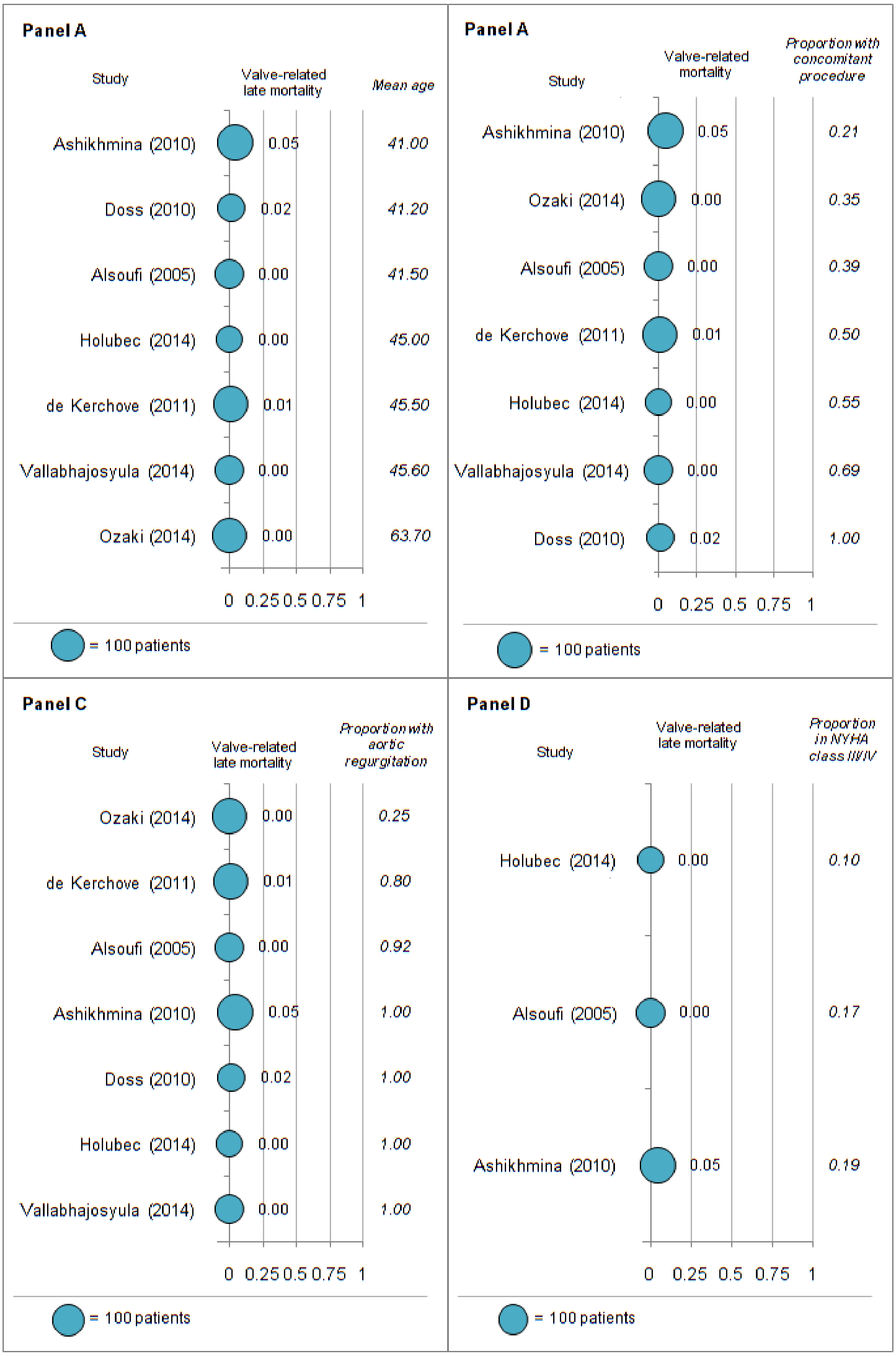

Figure S11: Effect of study-level baseline patient characteristics on valve-related reinterventions at follow-up

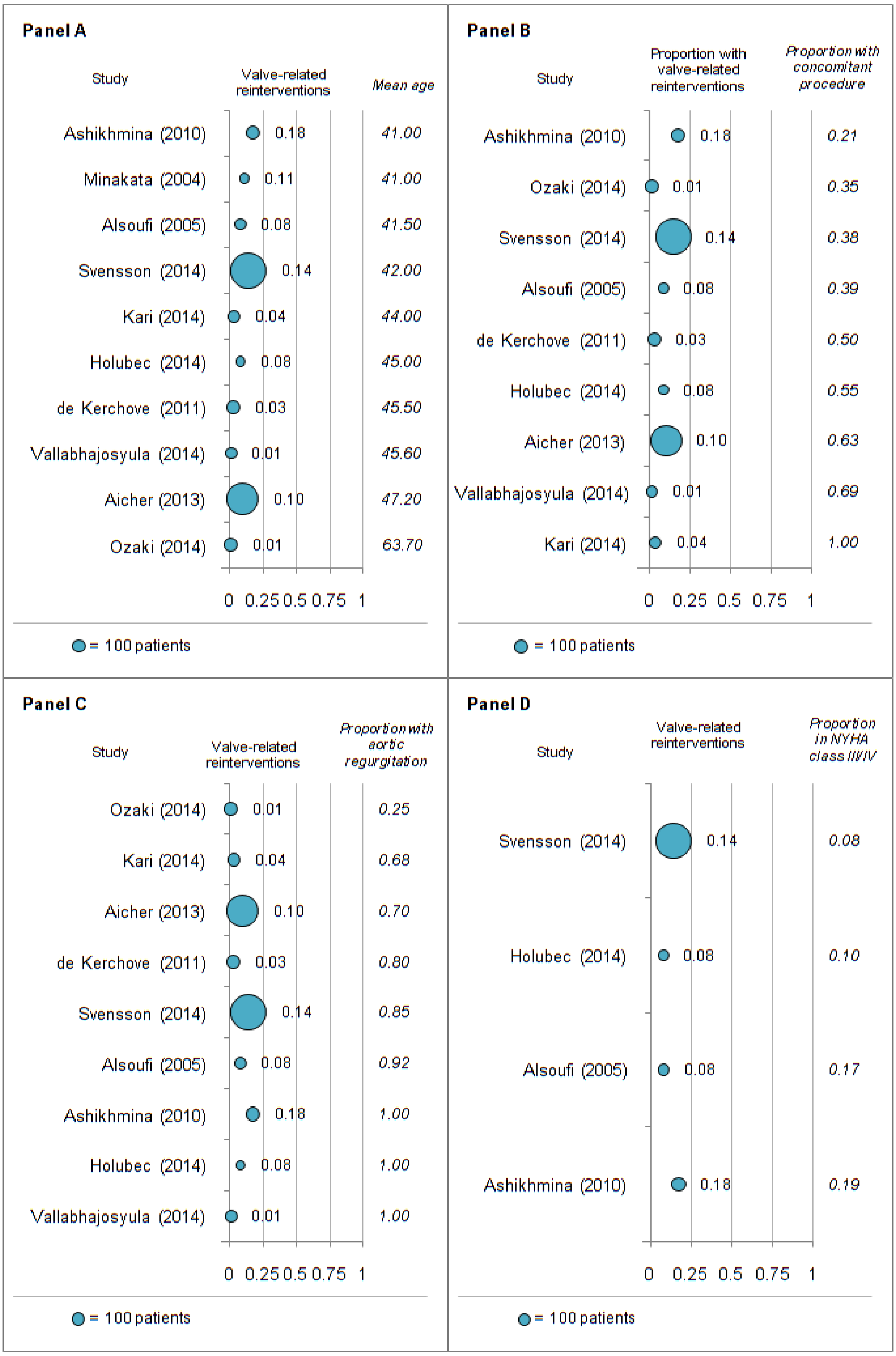

C. Included studies

Author	Publication	Location	Patients	Mean age (yrs)	Mean follow-up time (yrs)	Outcomes reported	Comments
Aicher (2013)	Aicher D, Schneider U, Schmied W, Kunihara T, Tochii M, Schafers HJ. Early results with annular support in reconstruction of the bicuspid aortic valve. J Thorac Cardiovasc Surg. 2013;145(3 Suppl):S30-4.	Germany	559	47.2	-	- 30-day survival<br>- Reinterventions on valve or prosthesis at f/u<br>- Reintervention-free survival at f/u	Comparison of valve repair with vs without annuloplasty. All patients received aortic valve repair and results were therefore extracted as one group.<br>No adjustment in analysis reported. Very limited information on baseline characteristics in the two groups reported.
Alsoufi (2005)	Alsoufi B, Borger MA, Armstrong S, Maganti M, David TE. Results of valve preservation and repair for bicuspid aortic valve insufficiency. The Journal of heart valve disease. 2005;14(6):752-8; discussion 8-9.	Canada	71	41.5	3.5	- Reoperation during index admission<br>- Neurologic event during index admission<br>- 30-day survival<br>- Survival at f/u<br>- Valve-related mortality at f/u<br>- Operated valve endocarditis at f/u<br>- Thrombosis, embolism, bleeding at f/u<br>- Reinterventions on valve or prosthesis at f/u (total)<br>- Reintervention-free survival at f/u	One cohort undergoing variety of valve repair techniques with ex-post analysis of repair and AV-sparing reimplantation or some outcomes. No adjustment reported. Baseline characteristics in the two groups not reported.

Author	Publication	Location	Patients	Mean age (yrs)	Mean follow-up time (yrs)	Outcomes reported	Comments
Ashikhmina (2010)	Ashikhmina E, Sundt TM, 3rd, Dearani JA, Connolly HM, Li Z, Schaff HV. Repair of the bicuspid aortic valve: a viable alternative to replacement with a bioprosthesis. J Thorac Cardiovasc Surg. 2010;139(6):1395-401.	United States	108	41	5.1	- Reoperation during index admission<br>- 30-day survival<br>- Survival at f/u<br>- Valve-related mortality at f/u<br>- Reinterventions on valve or prosthesis at f/u (total)	Case-control study matching 81 repair patients with replacement controls based on age, sex, and year of operation. Separate results only for limited outcomes.
de Kerchove (2011)	de Kerchove L, Boodhwani M, Glineur D, Vandyck M, Vanoverschelde JL, Noirhomme P, et al. Valve sparing-root replacement with the reimplantation technique to increase the durability of bicuspid aortic valve repair. J Thorac Cardiovasc Surg. 2011;142(6):1430-8.	Belgium	106	45.5	3.5	- Reoperation during index admission<br>- 30-day survival<br>- Valve-related mortality at f/u<br>- Neurologic event during index admission<br>- Operated valve endocarditis at f/u<br>- Reinterventions on valve or prosthesis at f/u (total)	Comparison of AV repair with subcommissural annuloplasty or AV-sparing root remodelling technique (Group 1) and AV repair with ventriculoaortic annuloplasty and AV-sparing root reimplantation (Group 2). Case-control study matching 53 pairs, based on maximum root diameter and preoperative AI.<br>The two groups were combined for this review.
Doss (2010)	Doss M, Risteski P, Sirat S, Bakhtiary F, Martens S, Moritz A. Aortic root stability in bicuspid aortic valve disease: patch augmentation plus reduction aortoplasty versus modified David type repair. European journal of cardio-thoracic surgery : official journal of the European Association for Cardio-thoracic Surgery. 2010;38(5):523-7.	Germany	66	41.2	5.1	- Reoperation during index admission<br>- 30-day survival<br>- Valve-related mortality at f/u<br>- Operated valve endocarditis at f/u	Comparison between two types of valve repair (patch augmentation plus reduction aortoplasty; patch augmentation plus modified David type repair). No adjustment reported.<br>Groups were combined for data extraction to obtain large enough sample size (>50 patients).

Author	Publication	Location	Patients	Mean age (yrs)	Mean follow-up time (yrs)	Outcomes reported	Comments
Holubec (2014)	Holubec T, Zacek P, Jamaliramin M, Emmert MY, Tuna M, Nedbal P, et al. Valve Cuspidity: A Risk Factor for Aortic Valve Repair? Journal of Cardiac Surgery. 2014;29:585-92.	Czech Republic	60	45	2	- 30-day survival<br>- Survival at f/u<br>- Valve-related mortality at f/u<br>- Operated valve endocarditis at f/u<br>- Thrombosis, embolism, bleeding at f/u<br>- Reinterventions on valve or prosthesis at f/u (total)	Comparison bicuspid vs tricuspid valve pathology.<br><br>Includes 3 patients with unicuspid valves which match classification for bicuspid valves and were therefore included in the bicuspid patient group.
Kari (2014)	Kari FA, Kvitting JPE, Stephens EH, Liang DH, Merk DR, Fischbein MP, et al. Tirone David procedure for bicuspid aortic valve disease: Impact of root geometry and valve type on mid-term outcomes. Interactive cardiovascular and thoracic surgery. 2014;19(3):375-81.	United States	85	44	2.6	- Operated valve endocarditis at f/u<br>- Reinterventions on valve or prosthesis at f/u (total)	Comparison of different types of BAV. Grouping not relevant for the review and data were therefore extracted together.
Minakata (2004)	Minakata K, Schaff HV, Zehr KJ, Dearani JA, Daly RC, Orszulak TA, et al. Is repair of aortic valve regurgitation a safe alternative to valve replacement? J Thorac Cardiovasc Surg. 2004;127(3):645-53.	United States	54	41	4.2	- Operated valve endocarditis at f/u<br>- Reintervention-free survival at f/u<br>- Reinterventions on valve or prosthesis at f/u (total)	
Ozaki (2014)	Ozaki S, Kawase I, Yamashita H, Uchida S, Nozawa Y, Takatoh M, et al. Reconstruction of bicuspid aortic valve with autologous pericardium - Usefulness of tricuspidization. Circulation Journal. 2014;78(5):1144-51.	Japan	102	63.7	2.0	- 30-day survival<br>- Survival at f/u<br>- Valve-related mortality at f/u<br>- Operated valve endocarditis at f/u<br>- Thrombosis, embolism, bleeding at f/u<br>- Reinterventions on valve or prosthesis at f/u (total)<br>- Reintervention-free survival at f/u	

Author	Publication	Location	Patients	Mean age (yrs)	Mean follow-up time (yrs)	Outcomes reported	Comments
Svensson (2014)	Svensson LG, Al Kindi AH, Vivacqua A, Pettersson GB, Gillinov AM, Mihaljevic T, et al. Long-term durability of bicuspid aortic valve repair. Ann Thorac Surg. 2014;97(5):1539-47; discussion 48.	United States	728	42	9	- Neurologic event during index admission<br>- 30-day survival<br>- Survival at f/u<br>- Reinterventions on valve or prosthesis at f/u (total)<br>- Reintervention-free survival at f/u	
Vallabhajosyula (2014)	Vallabhajosyula P, Komlo C, Szeto WY, Wallen TJ, Desai N, Bavaria JE. Root stabilization of the repaired bicuspid aortic valve: subcommissural annuloplasty versus root reimplantation. Ann Thorac Surg. 2014;97(4):1227-34.	United States	71	45.6	3.2	- Reoperation during index admission<br>- Neurologic event during index admission<br>- 30-day survival<br>- Survival at f/u<br>- Valve-related mortality at f/u<br>- Operated valve endocarditis at f/u<br>- Thrombosis, embolism, bleeding at f/u<br>- Reinterventions on valve or prosthesis at f/u (total)<br>- Reintervention-free survival at f/u	Comparison of two types of valve repair (repair and subcommissural annuloplasty; repair and aortic root reimplantation). No adjustment reported. The two groups were combined for this review.

D. Search strategy

MEDLINE via PubMed, 30/10/2014
Jan 1990 – Oct 2014

1	(Bicuspid aortic valve) OR bicuspid[Title/Abstract]	2,929
2	(Aortic valve repair) OR (Aortic valve preservation) OR (Aortic valve reconstruction) OR Repair[Title/Abstract] OR Remodelling[Title/Abstract] OR Remodeling[Title/Abstract] OR (Aortic valvotomy) OR (Aortic valvulotomy) OR tricuspidization[Title/Abstract] OR Commissurotomy[Title/Abstract] OR (valve sparing aortic root replacement) OR (yacoub[Title/Abstract] AND procedure[Title/Abstract]) OR (David[Title/Abstract] AND procedure[Title/Abstract])	299,458
3	(surgical valve replacement) OR (aortic valve replacement) OR "Aortic Valve/surgery"[MAJR] OR "Heart Valve Prosthesis Implantation"[Mesh] OR "Heart Valve Prosthesis"[Mesh] OR Prosthesis[Title/Abstract] OR Implant*[Title/Abstract] OR Graft*[Title/Abstract]	569,444
4	1 AND (2 OR 3)	1,232
5	Letter[ptyp] OR Editorial[ptyp] OR Comment[ptyp]	26
6	4 NOT 5	1,206
7	Limit to: Human; English	894
8	Limit to: Publication date from 1990/01/01	829

EMBASE, 30/10/2014
Jan 1990 – Oct 2014

1	'bicuspid aortic valve'/exp OR (bicuspid aortic valve)	3,930
2	'aortic valve repair'/exp OR (aortic AND valve AND repair) OR (aortic AND valve AND preservation) OR (aortic AND valve AND reconstruction) OR ('aortic valve' NEAR/3 repair):ab,ti OR ('aortic valve' NEAR/3 remodelling):ab,ti OR ('aortic valve' NEAR/3 remodeling):ab,ti OR (aortic NEAR/3 valvotomy):ab,ti OR (aortic NEAR/3 valvulotomy):ab,ti OR tricuspidization:ab,ti OR commissurotomy:ab,ti OR 'valve sparing aortic root replacement' OR (yacoub AND procedure):ab,ti OR (david AND procedure):ab,ti	12,696
3	'aortic valve replacement'/exp OR 'aortic valve prosthesis'/exp OR (surgical:ab,ti AND valve:ab,ti AND replacement:ab,ti) OR (aortic:ab,ti AND valve:ab,ti AND replacement:ab,ti)	33,434
4	1 AND (2 OR 3)	1,682
5	4 AND [humans]/lim AND [english]/lim NOT ([editorial]/lim OR [letter]/lim)	1,359
6	Limit to: Publication date from 1990/01/01	1,325
7	Exclude MEDLINE records	432

Cochrane Library, 30/10/2014
1990 – 2014

1	(Bicuspid aortic valve) or bicuspid	736
2	(Aortic valve repair) OR (Aortic valve preservation) OR (Aortic valve reconstruction) OR Repair OR Remodelling OR Remodeling OR (Aortic valvotomy) OR (Aortic valvulotomy) OR tricuspidization OR Commissurotomy OR (valve sparing aortic root replacement) OR (yacoub procedure) OR (David procedure)	10,769
3	(MeSH descriptor: [Heart Valve Prosthesis] explode all trees) OR (surgical valve replacement) OR (aortic valve replacement) OR Prosthesis OR Implant* OR Graft*	34,005
4	1 AND (2 OR 3)	131
5	Limit to: Publication date from 1990	130

	(12 Cochrane reviews; 3 other reviews; 114 trials; 1 economic evaluation)	

<u>CINAHL Plus via EBSCOHost, 30/10/2014</u>
Jan 1990 – Oct 2014

1	(AB bicuspid aortic valve) OR (TI bicuspid aortic valve)	289
2	(TI aortic valve repair) OR (AB aortic valve repair) OR (TI Aortic valve preservation) OR (AB Aortic valve preservation) OR (TI Aortic valve reconstruction) OR (AB Aortic valve reconstruction) OR (AB Repair OR TI Repair) OR (AB Remodelling OR TI Remodelling) OR (AB Remodeling OR TI Remodeling) OR (AB Aortic valvotomy OR TI Aortic valvotomy) OR (AB Aortic valvulotomy OR TI Aortic valvulotomy) OR (AB tricuspidization OR TI tricuspidization) OR (AB Commissurotomy OR TI Commissurotomy) OR (AB valve sparing aortic root replacement OR TI valve sparing aortic root replacement) OR (AB yacoub procedure OR TI yacoub procedure) OR (AB david procedure OR TI david procedure)	23,058
3	(MH "Heart Valve Prosthesis") OR (AB surgical valve replacement OR TI surgical valve replacement) OR (AB aortic valve replacement OR TI aortic valve replacement) OR (AB Prosthesis OR TI Prosthesis) OR (AB Implant* OR TI Implant*) OR (AB graft* OR TI graft*)	55,195
4	1 AND (2 OR 3)	89
5	Limit to: Human; English; Publication date from 1990/01/01	38
6	Exclude MEDLINE records	1
